# Effects of baclofen on insular gain anticipation in alcohol-dependent patients — a randomized, placebo-controlled, pharmaco-fMRI pilot trial

**DOI:** 10.1007/s00213-022-06291-6

**Published:** 2022-12-20

**Authors:** Patricia Pelz, Alexander Genauck, Robert C. Lorenz, Torsten Wüstenberg, Carolin Wackerhagen, Katrin Charlet, Tobias Gleich, Olga Geisel, Andreas Heinz, Christian A. Müller, Anne Beck

**Affiliations:** 1 Department of Psychiatry and Neurosciences | CCM, Charité – Universitätsmedizin Berlin, Charitéplatz 1, 10117 Berlin, Germany; 2 Bernstein Center for Computational Neuroscience Berlin (BCCN), Unter Den Linden 6, 10099 Berlin, Germany; 3 Lise Meitner Group for Environmental Neuroscience, Max Planck Institute for Human Development, Lentzallee 94, 14195 Berlin, Germany; 4 Research Council Field of Focus IV, Core Facility for Neuroscience of Self-Regulation (CNSR), Heidelberg University, Hauptstr. 51, Building 3011, 69117 Heidelberg, Germany; 5 Section On Clinical Genomics and Experimental Therapeutics (CGET), National Institute On Alcohol Abuse and Alcoholism (NIAAA), National Institutes of Health (NIH), 10 Center Drive, Bethesda, MD 20892-1540 USA; 6 Epilepsy-Center Berlin-Brandenburg, Evangelisches Krankenhaus Königin Elisabeth Herzberge, Herzbergstr. 79, 10365 Berlin, Germany; 7 Health and Medical University, Campus Potsdam, Olympischer Weg 1, 14471 Potsdam, Germany

**Keywords:** Baclofen, Alcohol use disorder, Functional magnetic resonance imaging (fMRI), Pharmaco-fMRI, Slot machine, Reward processing

## Abstract

**Rationale:**

One hallmark of addiction is an altered neuronal reward processing. In healthy individuals (HC), reduced activity in fronto-striatal regions including the insula has been observed when a reward anticipation task was performed repeatedly. This effect could indicate a desensitization of the neural reward system due to repetition. Here, we investigated this hypothesis in a cohort of patients with alcohol use disorder (AUD), who have been treated with baclofen or a placebo. The efficacy of baclofen in AUD patients has been shown to have positive clinical effects, possibly via indirectly affecting structures within the neuronal reward system.

**Objectives:**

Twenty-eight recently detoxified patients (13 receiving baclofen (BAC), 15 receiving placebo (PLA)) were investigated within a longitudinal, double-blind, and randomized pharmaco-fMRI design with an individually adjusted daily dosage of 30–270 mg.

**Methods:**

Brain responses were captured by functional magnetic resonance imaging (fMRI) during reward anticipation while participating in a slot machine paradigm before (t1) and after 2 weeks of individual high-dose medication (t2).

**Results:**

Abstinence rates were significantly higher in the BAC compared to the PLA group during the 12-week high-dose medication phase. At t1, all patients showed significant bilateral striatal activation. At t2, the BAC group showed a significant decrease in insular activation compared to the PLA group.

**Conclusions:**

By affecting insular information processing, baclofen might enable a more flexible neuronal adaptation during recurrent reward anticipation, which could resemble a desensitization as previously observed in HC. This result strengthens the modulation of the reward system as a potential mechanism of action of baclofen.

**Trial registration:**

Identifier of the main trial (the BACLAD study) at clinical.gov: NCT0126665.

**Supplementary Information:**

The online version contains supplementary material available at 10.1007/s00213-022-06291-6.

## Introduction

A hallmark of the neurobiological basis of addictive disorders is an altered recruitment of the brain’s reward system (Adinoff 2004) during processing of *drug-associated* as well as *non-drug associated cues* in patients with alcohol use disorder (AUD) (Bruguier et al. 2008; Lorenz et al. 2014). Specifically, an increased salience attribution to *alcohol-associated cues* (Grüsser et al. 2004; Heinz 2002; Robinson and Berridge 1993, 2000) and a concomitantly decreased salience attribution towards *non-alcohol associated cues* (e.g., monetary rewards) have been observed in AUD (Beck et al. 2009; Volkow et al. 2016; Wrase et al. 2007). Moreover, in patients with addictive disorders compared to healthy individuals (HC), reduced activation of striatal circuits during the presentation of *non-drug-associated* rewards has been described in a meta-analysis (Luijten et al. 2017). During *reward anticipation*, an increased dopaminergic firing rate (Di Chiara 1997) is demonstrated in the ventral tegmental area (VTA) (di Volo et al. 2018) prior to reward delivery or outcome (Schultz et al. 1997). Via mesolimbic and mesocortical pathways, the VTA is closely connected with other essential regions of the reward network including the prefrontal cortex (PFC), ventral striatum (VS), putamen, and insula (Bruguier et al. 2008). The insula is highly relevant for addictive behavior, as insular lesions have been shown to immediately abolish tobacco dependence (Bechara 2001). The insula is known to be specifically involved in the processing of uncertainty and proprioceptive self-awareness as well as in avoidance behavior, risky decision-making, gambling, and purchasing scenarios (Bruguier et al. 2008; Lorenz et al. 2014; Tsakiris et al. 2007). Structurally, the insula is indirectly connected to striatal areas via projections to medial prefrontal areas (Haber and Knutson 2010). There is evidence for two functional insular sub-units primarily involved in (1) the subjective processing of emotionally relevant content (Singer et al. 2009) and (2) the processing of uncertainty (Huettel et al. 2005; Preuschoff et al. 2008). Both processes are induced by the here-used slot machine paradigm: when performing the task, a potential win option represents an emotionally salient and arousing event (e.g., two congruent cylinders; C1 = C2) (Craig 2009), which is often accompanied by a perception of uncertainty during gain anticipation because the outcome of the third and final cylinder is uncertain (C1 = C2 = C3 or ≠ C3) (Lorenz et al. 2014). Past research showed in HC, playing a slot machine lead to high fronto-striatal activation (Knutson et al. 2001; Lorenz et al. 2015a, b; Luijten et al. 2017), whereas playing the slot machine repeatedly was associated with decreased fronto-striatal brain activation ((Lorenz et al. 2015a, b). The authors concluded that specifically, a reduction in salience and uncertainty at t2 might contribute to a desensitization of the reward system that is accompanied by the observed reduction in fronto-striatal activation. In other words, this desensitization of the reward system seems to represent an adaptative mechanism which might be impaired in AUD. In this study, we focused on the described comparison between gain anticipation (two congruent cylinders; C1 = C2) and no-gain anticipation trials (two incongruent cylinders; C1 ≠ C2 (Lorenz et al. 2014) when performing the task a second time (t2).

Regarding pharmacological treatment options for AUD, baclofen — a gamma-amino-butyric acid-_B_ (GABA-_B_) receptor agonist — received attention as a potential addition (Agabio et al. 2018) to currently approved pharmacological interventions (i.e., naltrexone, acamprosate, and disulfiram) (Shen 2018), since the latter showed only limited effectiveness (Jonas et al. 2014). So far, baclofen effects for the treatment of AUD are still controversial.

To date, three recent meta-analyses assessed the efficacy of baclofen of which two found evidence for significant efficacy of baclofen in AUD with a higher percentage of abstinent patients at the study end and a longer time to lapse compared to placebo (Pierce et al. 2018; Rose and Jones 2018). In contrast, Bschor and colleagues (2018) found no superiority of baclofen versus placebo. However, an expert consortium suggests individual titration in patients with the treatment goal of achieving abstinence and/or reduced drinking (Agabio et al. 2018), taking into account the individual severity and history of the disease (Shen 2018).

It is assumed that AUD leads to a downregulation of the GABAergic system and simultaneously to an alteration of fronto-striatal information processing (Beck et al. 2012; Goldstein and Volkow 2002, 2011). Thus, baclofen as a GABAergic agonist, might have the ability to interfere indirectly with fronto-striatal circuits. Regarding the neuronal mode of action, a preclinical study by Fadda and colleagues showed that baclofen suppressed the alcohol-induced dopamine release in rodents’ nucleus accumbens (Nacc), assuming an indirect mechanism via inhibitory GABA-_B_ receptors in VTA and the associated suppression of dopaminergic signaling towards the Nacc or the VS (Fadda et al. 2003). In humans, baclofen affects neural reward processing (Beck et al. 2018; Boehm et al. 2002), although the exact mechanism of action remains to be elucidated (Müller et al. 2015). Studies investigating nicotine dependence observed that baclofen decreased resting state blood flow in the insula and VS (Franklin et al. 2011, 2012) while in cocaine-dependent patients, baclofen diminished activation in response to subliminal cocaine cues in bilateral VS, ventral pallidum, amygdala, midbrain, and orbitofrontal cortex (OFC) — regions known to be involved in motivated behavior (Volkow et al. 2016; Young et al. 2014). These findings support the hypothesis that baclofen acts in similar ways with respect to different drugs of abuse. Additionally, a recent pharmaco-fMRI study in patients with AUD found increased abstinence rates together with high-dose baclofen-induced functional reductions of alcohol cue-associated brain responses in OFC, amygdala, and VTA — areas known to be involved in the processing of salient stimuli (Beck et al. 2018). In line, another pharmaco-fMRI study showed decreased alcohol-associated cue-elicited BOLD signal mostly in frontal areas, i.e., precentral gyri and ACC in patients with AUD treated with 75 mg/daily baclofen dosage compared to placebo-treated patients (Logge et al. 2019).

In our current study, we investigated whether baclofen modulates the neural activity during rewarding cues in general. The applied slot machine paradigm (Lorenz et al. 2014) enables the assessment of alterations in the fronto-striatal network during the anticipation of monetary (*non-alcohol-associated*) rewards. In particular, based on the former findings in HCs (Lorenz et al. 2015a, b), we hypothesize that baclofen decreases (“desensitizes”) neuronal activation in the fronto-striatal network including the insula in patients with AUD when performing the slot machine a second time, representing a more flexible adaptation towards reduced emotional arousal or uncertainty. To test whether our observed effects were related to the administration of baclofen, we investigated associations between brain response and baclofen blood serum level in exploratory analyses.

## Experimental procedures

### Study description

The study was a preregistered, randomized, double-blind, and placebo-controlled pharmacological trial with 56 AUD patients ((clinical.gov: NCT01266655; published BACLAD-study; (Müller et al. 2015) with an individual titration up to high-dose baclofen or placebo (range from 30 mg/day up to 270 mg/day) for 12 weeks and an embedded functional magnetic resonance imaging part (fMRI) with two scanning sessions: one at baseline, before starting the titration (t1) and one recurrent after 2 weeks of individual high-dose intake (t2). Placebo capsules contained mannitol (99.5%) and silicium dioxide (0.5%). Patients were titrated from week 1 to 4 until they reached their individual dosage, followed by a 12-week stable individual high-dose phase, and were tapered in the same way as they were titrated. We here report data of the fMRI sample (*n* = 28), who participated at t1 and t2. All patients were recruited during in- and outpatient detoxification treatment and were randomly assigned to one of the two study groups after completion of detoxification. This study was conducted in accordance with the Declaration of Helsinki and approved by the ethics committee of Charité—Universitätsmedizin Berlin. The BACLAD study (Müller et al. 2015) was approved by the ethics committee of the state of Berlin and the Federal Institute for Drugs and Medical Devices (BfArM). All patients gave written fully informed consent for participation.

### Participants

We included patients with AUD between 18 to 65 years, with a weekly minimum of two heavy drinking days (defined as drinks per day: men ≥ 5/day, women ≥ 4/day; 1 standard drink equals 12 g absolute alcohol). We excluded other psychiatric axis I-disorders ((SCID-interview, (Fydrich 1997), gambling disorders and substance dependences other than nicotine dependence, as well as AUD patients with abstinence durations longer than 21 days and with irremovable ferromagnetic material (for detailed inclusion and exclusion criteria please see Beck et al. (2018) and Müller et al. (2015)). No study participant had used baclofen before.

**Fig. 1:**
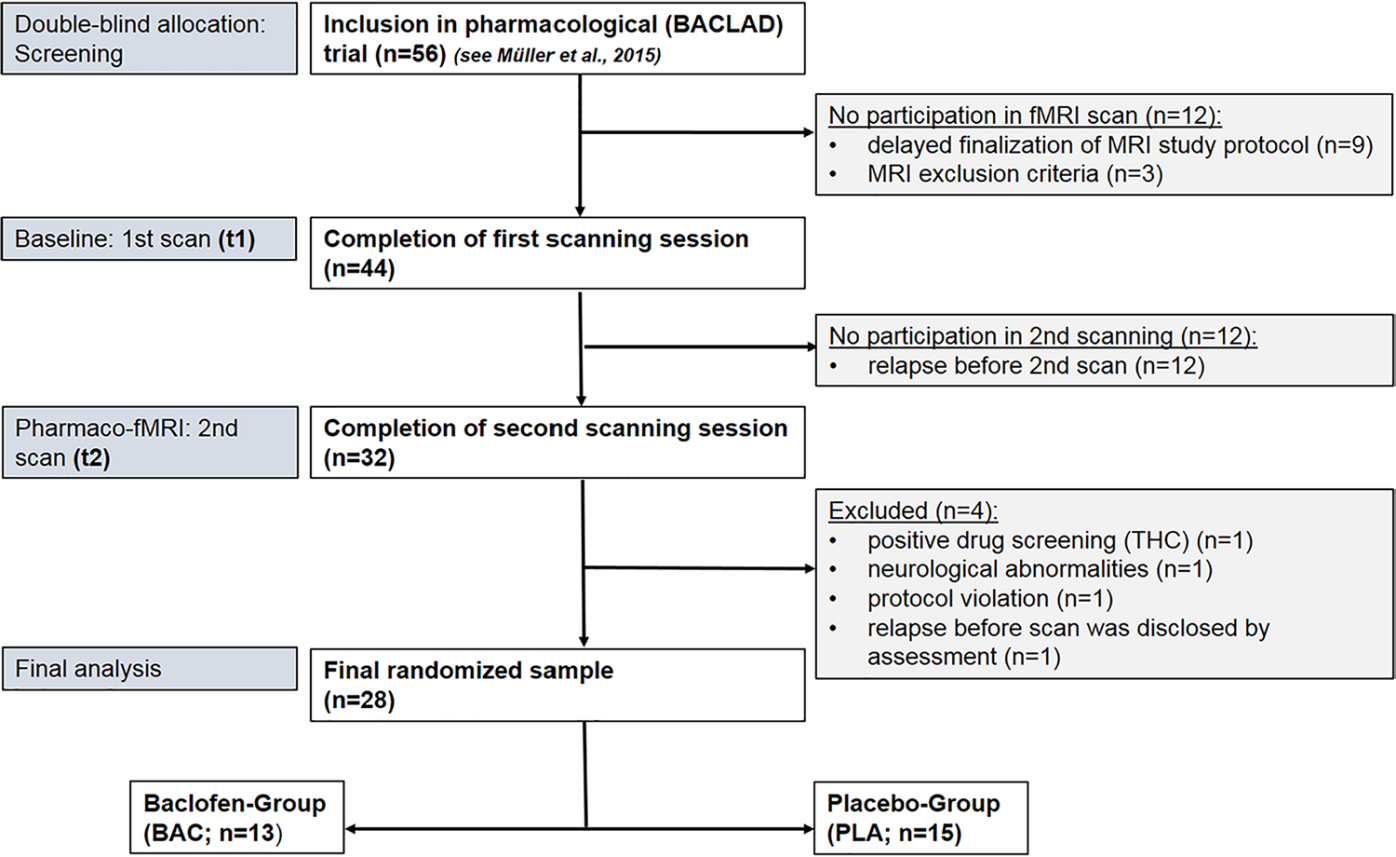
CONSORT flow diagram: overview of the study allocation, number of participants and drop-outs

Finally, from 56 patients who participated in the BACLAD trial (Müller et al. 2015), a total of 28 (13 BAC; mean age = 47.54 ± SD = 0.83 and 15 PLA; mean age = 47.0 ± SD = 0.26, *p* = . 861; see Table 1) were included in our analysis. Blood serum levels of the study medication were assessed in the BAC group at t2. One blood sample has not been analyzed due to missing data at this time point. When replacing the missing value with the median, the result remained significant. A detailed overview of the study allocation and reasons for exclusion is given in Fig. 1.

**Table 1:** Differences between groups in demographic and clinical data

Variable and time (t1 or t2)	Groups				Statistics		
	Baclofen *n* = 13 (2 females)		Placebo *n* = 15 (6 females)		Permutation test		*CI*
	M	SD	M	SD	df	*p* value	
Descriptive parameters at baseline (t1)							
Age	47.54	7.83	47	7.26	26	.861	[− 5.21, 5.64]
Years of education	14.25	2.50	15.2	3	26	.891	[− 4.40, 4.61]
WST	104.75 ^A)^	14.22	101.8	9.01	25	.534	[− 4.68, 13.42]
Handedness (laterality quotient (%))	83.52	12.75	81.45	21.81	26	.777	[− 8.93, 16.75]
FTND (number of current smokers)	3.57 (8 smokers)	2.64	5.59 (9 smokers)	1.99	26	.154	
PY (years)	17.03	11.6	16.99	13.75	26	1	[− 10.22, 8.52]
Alcohol related parameters at baseline (t1) and during high-dose phase (t2)							
Duration of AUD (years); t1	17.38	11.76	11.27	8.54	26	.126	[− 1.27, 13.56]
Detoxifications (number); t1	3.92	5.11	2.47	1.64	26	.856	[− 1.24, 1.66]
Daily alcohol intake last 30 days (gram); t1	221.69	102.65	160^A)^	68.35	25	.071	[− 2.99, 123.13]
LDH (kilogram); t1	1165.33	872.85	615.24	477.24	26	.045*	[93302.81, 1,221,399,23]
Abstinent days prior to study inclusion (days); t1	13.69	4.15	11.8	4.02	26	.235	[− 1.03, 4.77]
Abstinence duration: start detox. to t2 (days); t2	50.23	6.78	53	4.88	26	.226	[− 6.94, 1.64]
Abstinence rate (%) 12 weeks high-dose phase	69.23% (9/13)	-	26.67% (4/15)	-	12	.030*^a^	
ADS; t1	15.31	5.38	15.47	4.9	26	.944	[− 3.33, 4.17]
OCDS-G, t1	20.15	6.28	21.93	8.51	26	.557	[− 7.46, 3.15]
OCDS-G; t2	13.08	9.95	14.95	12.78	26	.56	[− 7.30, 3.28]
Individual high-dose (mg/d/range)	152.31 (60–270)	79.81	252 (150–270)	38.95	26	< .001*	[− 142.62, − 50.46]
Baclofen serum level (µg/l)	762.34^A)^	381.13	-	-	-	-	-
Clinical parameters at baseline (t1) and during high-dose phase (t2)							
BDI-II sum score; t1	7.4^C)^	3.66	10.36^A)^	5.58	22	.164	[− 6.5, .65]
BDI-II sum score; t2	10.67^A)^	7.92	11.92^C)^	12.13	22	.781	[− 10.20, 5.79]
STAI-state sum score; t1	39.69	4.42	43.67	4.07	26	.02*	[− 7.31, − 1.13]
STAI-state sum score; t2	41.85	5	41.73	6.33	26	1	[− 3.82, 4.30]
BIS-11 sum score; t1	63.83^A)^	6.66	61.17^C)^	15.83	22	.609	[− 6.88, 11.70]
BIS-11 sum score; t2	65^B)^	8.5	61^A)^	11.24	23	.336	[− 3.38, 11.53]

*M*, mean; *SD*, standard deviation; *CI*, confidence interval of effect statistic (95% lower, upper); *df*, degree of freedom; *p*-value was permuted, except: ^a^exact chi-square test; ^b^number of abstinent patients *significant differences; *t1*, baseline scanning session; *t2*, second scanning session; number of missing values due to technical error or refusal by subject to answer, replaced by median of respective group, in detail: ^A)^1 missing case, ^B)^2 missing cases, ^C)^3 missing cases; *AUD*, alcohol use disorder; *WST*, IQ measured by German version of vocabulary test (“Wortschatztest”); handedness measured by Edinburgh Handedness Inventory; *FTND*, Fagerström test for nicotine dependence; *PY*, pack years of nicotine use; *LDH*, life time drinking questionnaire; *ADS*, severity of dependence measured by alcohol dependence scale; *OCDS-G*, craving measured by obsessive compulsive drinking scale; *BDI-II*, depression measured by Beck’s depression inventory II; *STAI*, state anxiety measured by Spielberger’s state trait anxiety inventory; *BIS-11*, impulsivity measured by Barrat impulsiveness scale 11

After patients terminated medically supervised detoxification, groups did not differ significantly in abstinent days prior to study inclusion. All patients were right-handed as confirmed by the Edinburgh Handedness Inventory (Oldfield 1971). Intellectual premorbid capacity was measured by the German vocabulary test “Wortschatztest” (WST) (Metzler and Schmidt 1992). Nicotine dependence was permitted and assessed by the Fagerström questionnaire for nicotine dependence (FTND) (Heatherton et al. 1991). For *alcohol-related measures*, lifetime total alcohol consumption in kilogram was evaluated by the life time drinking history (LDH) interview (Skinner and Sheu 1982). The amount of daily absolute alcohol consumption in gram was recorded for the last 30 days before the baseline scan via timeline followback (TLFB) (Sobell and Sobell 1992). The severity of illness was assessed by the alcohol dependence scale (ADS) (Skinner and Horn 1984) for the last 12 months. Craving was measured using the obsessive compulsive drinking scale (OCDS-G) (Mann and Ackermann 2000) for the last 7 days before t1 and the last 14 days before t2. *Clinical and personality measures* included the state-trait anxiety inventory (STAI-state) (Laux et al. 1981), the beck depression inventory (BDI-II) (Beck et al. 1961), and the Barratt impulsiveness scale (BIS-11) (Patton et al. 1995) each at t1 and t2. Detailed sample characteristics are provided in Table 1.

All behavioral, clinical, and sociodemographic data to describe the two groups (BAC, PLA) were analyzed using non-parametrical linear model with the group as between subject-factor tests with permutation-based alpha-error (*p*-value) estimation (using R package lmPerm (https://cran.r-project.org/web/packages/lmPerm/index.html), with max. 1 million iterations and Ca = 0.001, i.e., package’s early stopping routine, namely when the estimated standard error of the estimated *p* is less than Ca*p). Permutation-based *p*-value computations are useful because they do not assume a certain distribution of the residuals and hence do not require a test for normal distribution of the residuals as would be the case for the *t*-tests for each covariate for sample characterization. The mean difference of each covariate (BAC minus PLA) was computed as well as the confidence interval of that difference using bootstrapping with 10,000 sampling repetitions (stratified per group) (using R package boot and bias-corrected 95% *CI* computation). Bootstrapping is useful to compute *CIs* without assuming any distribution of the covariates. Abstinence rates were analyzed by using the exact chi-square test, using R software (www.r-project.org).

### Slot machine paradigm

To investigate reward anticipation–related brain responses in the fronto-striatal reward system, we used a virtual slot machine paradigm with three moving cylinders (C1, C2, C3) with two different sets of fruits (cherries and lemons, or melons and bananas) at two time points (t1, t2) (Lorenz et al. 2014, 2015a, b). Stimuli were presented in a pseudo-randomized manner. The slot machine was programmed using Presentation software (Version 14.9, Neurobehavioral Systems Inc., Albany, CA, USA). The paradigm is a so-called passive reward task since participants have no influence or control regarding reward outcome as they would have in performance-driven reward tasks (e.g., monetary incentive delay tasks, MID, Knutson et al. 2000; Richards et al. 2013). Prior to the MR-scanning session, patients were familiarized with the task for 5 trials in the scanner. During the experimental phase, patients were asked to play the slot machine 60 times with a wager of 0.10 € per trial; each participant had a total wager amount of 6.00 € before starting the task. The aim in each trial was to get three equal fruit cylinders in a horizontal line (C1 = C2 = C3; win trial), which was rewarded with 0.50 €. In all other cases (early loss C1 ≠ C2 and late loss C1 = C2 ≠ C3), patients lost 0.10 € additionally to the wager of 0.10 €.

**Fig. 2:**
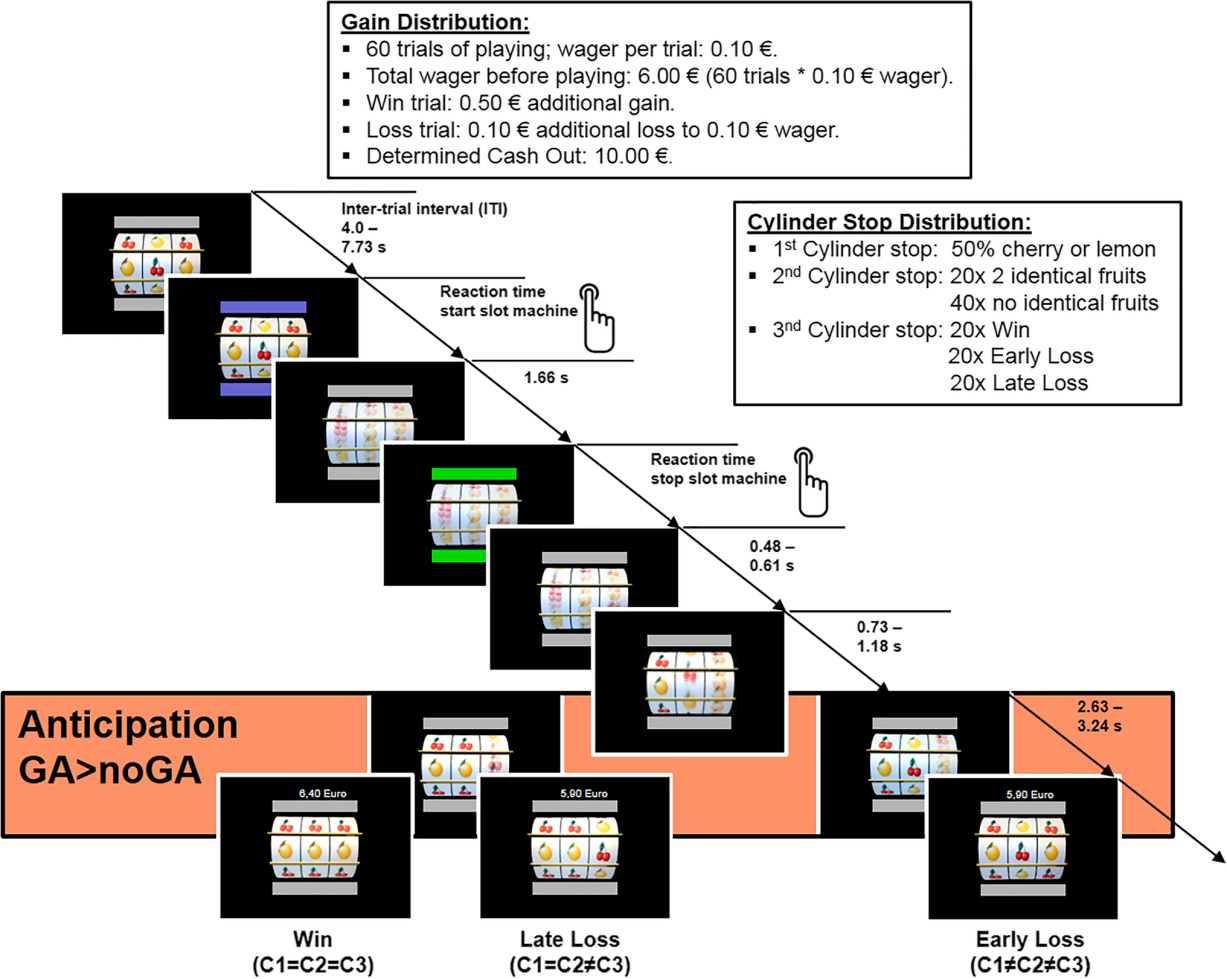
Slot machine paradigm

Each trial started with gray color bars indicating the inactive state of the cylinders, which turned blue to notify participants of the start signal. Participants started the slot machine by pressing the start button with their right index finger. The three cylinders were then accelerated using an exponential profile from the left to the right cylinder. In a total of 1.66 s after pressing the start button, the maximum speed was reached, and the color bar turned from gray to green — the signal to stop the machine during the next 4 s via the same button press. After pressing the button, the cylinders stopped from left to right. The left (first) cylinder stopped after 0.48 to 0.61 s, the middle cylinder stopped after an additional 0.73 to 1.18 s, and the right (and third) cylinder stopped after another 2.63 to 3.24 s. The stop of the right cylinder terminated the trial and the current win/loss and total amount of money (minus the 0.10 € wager) was presented above the slot machine. After a variable inter-trial interval (ITI) between 4.0 and 7.43 s, the color bar turned from gray to blue again, indicating a new trial. In this publication, we analyzed the phase of *gain anticipation* which indicates the time period between the stop of the second and third cylinder: Two equal fruit cylinders (C1 = C2) indicate the possibility to further win *(gain anticipation; GA)* while two unequal cylinders (C1 ≠ C2) indicate an upcoming loss *(no gain anticipation; noGA)*. To guarantee an equal number of trials for each of the three conditions (win: C1 = C2 = C3, 20 trials; early loss: C1 ≠ C2 ≠ C3 or = C3, 20 trials; late loss: C1 = C2 ≠ C3, 20 trials), the win probabilities were equalized to *p* = 0.33. Due to the self-paced nature of the experiment, the total execution time ranged between 12 and 14 min. In this time period, 360 to 420 functional brain images were acquired (see Fig. 2).

Highlighted in light orange: exemplary anticipation phase with 2 lemons indicating possible gain (GA) vs. 1 lemon and 1 cherry indicating no gain possible (noGA).

### fMRI data acquisition and scanning procedure

FMRI data acquisition was carried out using a 3 Tesla TIM Trio MRI scanner (Siemens Healthineers AG, Erlangen, Germany) equipped with a 12-channel phased array head coil at the Berlin Center for Advanced Neuroimaging (BCAN, Charité – Universitätsmedizin Berlin). The goggle system FA-nnl from Nordic Neurolab, with a resolution of 800 × 600 (http://www.nordicneurolab.com/products/VisualSystem.html) was used for stimulus presentation. Start and stop commands were given via a 4-button fiber optic response box (fORP, Current Design Inc.). Whole head functional brain images were acquired using a T2*-weighted gradient echo planar imaging sequence (GE–EPI) with 33 axial oriented slices (descending acquisition order, time to repeat (TR) = 2000 ms, time to echo (TE) = 30 ms, field of view (FoV) = 192 × 192, flip angle = 78°, matrix size = 64 × 64, isometric voxel size = 3 × 3 × 3mm^3^). For anatomical reference and optimal warping of functional brain images into stereotactical standard space, a T1-weighted magnetization prepared gradient-echo sequence (MPRAGE) was collected before functional imaging with 192 sagittal slices, T = 1900 ms, TE = 2.52 ms, flip angle = 9°, FoV = 256 × 256, matrix size = 256 × 256, isometric voxel size = 1 × 1 × 1 mm^3^.

### Image processing

Image preprocessing and statistical analyses were conducted using the Statistical Parametric Mapping software package (SPM12, Wellcome Department of Imaging Neuroscience, London, UK, http://www.fil.ion.ucl.ac.uk/spm/software/spm12/). Before preprocessing, DICOM data were converted into Nifti format via MRIConvert (University of Oregon). Next, imaging data were manually inspected for image artifacts, the origin was set to the anterior commissure, and the images were oriented to roughly match the orientation of the used brain template. Additionally, spatial matching between functional and anatomical scans was checked via MRIcron (www.mricro.com).

After these preparatory steps, EPIs were corrected for acquisition time delay as well as head motion and the MPRAGE was co-registered to the mean EPI and segmented and transformed into the stereotactic standard space as defined by the ICBM (International Consortium for Brain Mapping) brain atlas implemented in SPM12 (http://www.loni.usc.edu/research/atlases) with the unified segmentation approach developed by Ashurner and Friston (2005). Using linear and nonlinear parameter estimates from this step, EPIs were warped and resampled with a voxel size of 3 × 3 × 3 mm^3^. Finally, EPIs were spatially smoothed with a moving isotropic 3D Gaussian kernel of 7 mm full width at half maximum.

### Modeling of individual brain responses

Data were analyzed voxel-wise within the framework of the Generalized Linear Model (GLM) in a mass univariate manner. On single subject level, we modelled periods of anticipatory brain activity by means of box-car functions with an onset at the stop of the second cylinder and a width equal to the time gap between the second and third cylinder stop. To model the BOLD responses, these functions were convolved with the canonical hemodynamic response function (hrf) as implemented in SPM12 and served as regressors of interest in single-subject models. Additionally, regressors of no interest were constructed in the same way for the win, late loss, and early loss. On the single-subject level, the model contained separate regressors for gain anticipation (C1 = C2) and no gain anticipation (C1 ≠ C2) as well as the following regressors of no interest: gain (C1 = C2 = C3), loss (C1 = C2 ≠ C3), early loss (e.g., (C1 ≠ C2 = C3), button presses (after color bar changed to blue as well as green), visual flow (rotation of the wheels), and the six rigid body movement parameters. In addition, start and stop button presses were modelled by means of stick functions and convolved with the hrf. To account for the different amount in visual flow, we used the number of cylinders in motion convolved with the hrf as a related proxy (Lorenz et al. 2015a, b). Nuisance variance in voxel-time series caused by susceptibility × motion interactions was modelled by means of the six motion parameter estimates derived from motion correction. Finally, to model the mean signal within the session, a constant was added to the GLM. After band-pass filtering and restricted maximum likelihood fit of the model to the data, two contrasts were calculated (Wiers et al. 2015). *Contrast 1:* gain anticipation at baseline (t1) ([GA > noGA] before treatment). This includes gain possible (e.g., C1 = C2) and no gain possible (C1 ≠ C2). *Contrast 2:* gain anticipation at baseline minus gain anticipation after 2 weeks of individual high-dose pharmacological treatment [GA > noGA at t2 – GA > noGA at t1].

### Statistical group analysis

On a group level, *t*-tests were used to calculate gain anticipation before treatment in both groups *(effect of task) *and between groups *(effect of treatment)*. In detail, for analyzing the *effect of task* before treatment, we used a one-sample *t*-test for contrast 1 for the whole sample (*n* = 28). Only the effects passing a statistical threshold of *p* < 0.05 family-wise error (FWE) whole brain corrected at the cluster level were considered for report and discussion and were specified in MNI coordinates in *x*, *y*, and *z* dimensions. We additionally assessed potential group differences using a two-sample *t*-test. For analyzing the *effect of treatment,* we conducted a two-sample-*t*-test using contrast 2 to calculate differences in gain anticipation between groups before and after treatment: [(GA > noGA (t2-t1) _PLA_] > [(GA > noGA) (t2-t1) _BAC_] (Wiers et al. 2015). Only the effects passing a statistical threshold of *p* < 0.001 family-wise error (FWE) corrected at the cluster level were considered for report and discussion.

Due to higher anxiety scores (STAI-state) and higher amounts of alcohol consumed over a lifetime (LDH) prior to treatment in patients receiving baclofen, the robustness of fMRI results was tested by introducing those variables as covariates of no interest into the model (Miller and Chapman 2001). Since our results remained robust after adjusting for both potential confounding variables, we followed authors who recommended not to co-vary baseline group differences (de Boer et al. 2015; Miller and Chapman 2001). De Boer and colleagues consider the adjustment of significant baseline differences “to be inappropriate and erroneous because it might ignore the fact that the prognostic strength of a variable is important even when there is an interest in adjusting to confounding effects” (de Boer et al. 2015). Nevertheless, we calculated the results with the covariates STAI-state and LDH (see the “Imaging result” section) but only discuss the non-covariate data in detail.

**Fig. 3:**
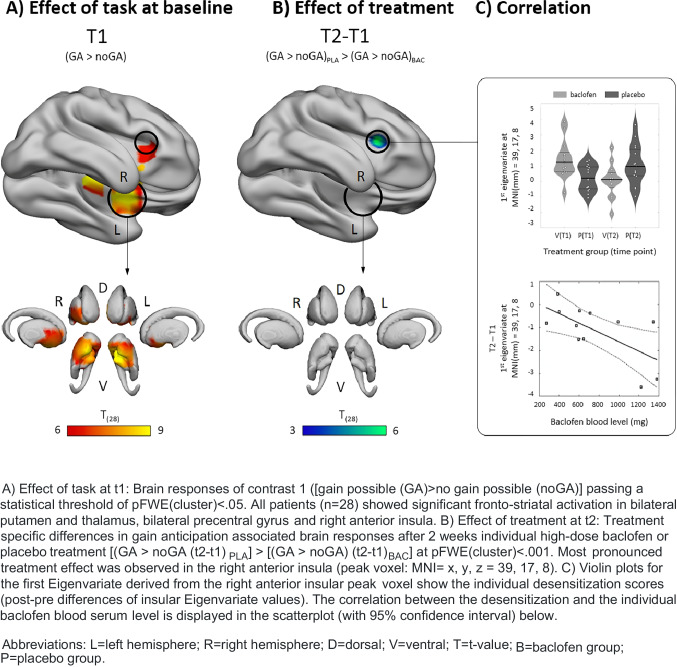
The effect of treatment and its correlation with baclofen blood serum level

Imaging results were anatomically identified using SPM implemented brain atlas “Neuromorphometrics”. In order to achieve a more precise result, the brain structure of the red nucleus was identified via Talairach Client and displayed in Fig. 3 using Surf Ice software (www.nitrc.org/projects/surfice). Violine plots were displayed using Matlab 2014b (www.mathworks.com).

### Correlation between insula response and baclofen blood serum level

We further extracted the first Eigenvariate within a sphere of 4 mm radius centered around the peak voxel (MNI = *x* = 39, *y* = 17, *z* = 8) of the most pronounced treatment effect (2-sample-*t*-test between BAC and PLA based on contrast 2 [(GA > noGA (t2-t1) _PLA_] > [(GA > noGA) (t2-t1) _BAC_] in the right anterior insula (see the “Results” section below). This Eigenvariate is the vector of beta coefficients of the T2-T1 contrast at peak voxel and thus reflects the post–pre difference in brain response being a measure of the desensitization effect as it has also been previously described (Lorenz et al. 2015a, b) during repeated slot machine gaming in the fronto-striatal network. To test whether this desensitization is influenced by the baclofen treatment, we explored its correlation with baclofen blood serum levels in the baclofen group using R software (www.r-project.org) as an additional biological marker. Serum levels of baclofen were assessed 2 weeks after reaching the individual high dose using liquid chromatography/mass spectrometry in blood serum.

We had a one-sided hypothesis (the higher the serum level, the higher the desensitization) and hence computed a one-sided *p*-value by bootstrapping (using R = 10,000 repetitions) the correlation coefficient *r* and checking in how many cases *n* is *r* <  = 0, hence *p*(*r* <  = 0) = *n*(*r* <  = 0) / R.

## Results

### Effect of medication on clinical outcomes

Demographic and clinical sample characteristics are provided in Table 1. *At t1,* groups differed significantly for STAI-state anxiety (p_permuted_ = 0.02) and lifetime drinking alcohol (LDH) amount in kilogram (p_permuted_ = 0.045). The groups did not differ significantly with respect to gender (chi-square: *p* = 0.150). *At t2*, PLA group (M = 252.0 mg/day ± SD = 38.95) showed significantly higher mean dosage than BAC group (M = 152.31 mg/day ± SD = 79.81; T_(28)_ =  − 4.101, *p* ≤ 0.001).

At the *end of the high-dose period* (10 weeks after the second MRI scanning), the BAC group showed significantly higher abstinent rates (*n* = 9/13, 69.23%) than in PLA group (*n* = 4/15, 26.67%; Fisher’s exact test p_one-sided_ = 0.030).

### Imaging results

#### Effects of task at baseline

At *t1* during gain anticipation, all patients (*n* = 28) showed fronto-striatal activation (Table 2). We observed the strongest effects in bilateral putamen left: T_(28)=_8.37, p_FWE(cluster)_ =  ≤ 0.000; right: T_(28)_ = 8.02, p_FWE(cluster)_ =  ≤ 0.001) and right thalamus (T_(28)=_7.55, p _FWE(cluster)_ = 0.001). When including covariates (STAI-state and LDH from t1) results remained significant in bilateral putamen left: T_(28)=_8.59, p_FWE(cluster)_ =  ≤ 0.000; right: T_(28)_ = 8.02, p_FWE(cluster)_ =  ≤ 0.001) and right thalamus (T_(28)=_7.83, p _FWE(cluster)_ = 0.001) (see supplementary material).

**Table 2:** Effect of task: brain regions

Brain regions	L/R	k	MNI			t	_FWE (cluster)_
			*x*	*y*	*z*		
Putamen	L	58	− 15	2	− 10	8.37	< .001
Putamen	R	46	18	5	− 10	8.02	< .001
Thalamus	R	16	9	− 22	-4	7.55	< .001
Red nucleus^A)^	L	4	− 6	− 25	− 10	6.52	.006
Anterior Insula	R	3	30	23	4	6.23	.009
Precentral gyrus	R	2	12	− 25	47	6.04	.013
Precentral gyrus	L	1	− 21	− 16	56	5.90	.022

*R*, right and; *L*, left hemisphere; *k*, cluster size; *x*, *y*, *z*, MNI Montreal neurobiological institute space; *t*, *t*-value; *p*, *p*-value at statistical threshold of *p* < .05 family-wise error (FWE) whole brain corrected at cluster level and reported via SPM implemented brain atlas “Neuromorphometrics.” ^A)^Reported via Talairach Client

There were no significant baseline group differences between the BAC and PLA groups.

#### Effect of treatment and correlation of insula response and baclofen blood serum level

At *t2*, the BAC group showed a significantly decreased activation in the right anterior insula compared to the PLA group (T_(28)=_5.30, MNI = *x* = 39, *y* = 17, *z* = 8, p_FWE(cluster)_ = 0.013), which can be interpreted as an indication of desensitization. This post–pre-difference of insular Eigenvariate values correlated significantly with baclofen blood serum level in the BAC group. In other words, the lower the functional insula activation, the higher the baclofen level in the blood serum. Additionally, the mean dosage of baclofen (mg/d) and baclofen blood serum levels were significantly correlated (*r* = 0.557, p_bootstrapped (one-sided)=_0.011). When including covariates (STAI-state (t2-t1) and LDH from t1), the results remained significant when covarying for these factors (T_(28)=_5.15, MNI = *x* = 33, *y* = 17, *z* = 5, p_FWE(cluster)_ = 0.005). The effect of treatment and its correlation with baclofen blood serum level is provided in Fig. 3.

## Discussion

The main finding of our study is a decrease in insula activation following baclofen versus placebo medication in a slot machine paradigm among patients with alcohol use disorder.

Prior to study inclusion, all patients showed high ADS and OCDS scores, high chronic alcohol consumption (LDH), and a high number of detoxifications, representing a severely affected sample of AUD patients. The BAC group showed even higher lifetime alcohol consumption (LDH) than the PLA group. In this context, it is important to note that Pierce and colleagues (2018) showed that those “ailing,” heavily drinking patients may specifically benefit from *high-dose* baclofen (> 60 mg/d) which held true for our present study.

On a clinical level, we observed significantly higher abstinence rates in the BAC compared to the PLA group, i.e., higher percentage of abstinent patients as well as longer abstinence durations during the 12-week high-dose phase, which has also been observed in our larger sample from our pharmacological trial (Müller et al. 2015). These results support the findings of the first studies by Addolorato and colleagues and also recent findings by de Beaurepaire and colleagues, who supported baclofen as a promising treatment approach, especially for patients with moderate to severe alcohol disorders (Addolorato et al. 2002; Colombo et al. 2004; de Beaurepaire et al. 2019). A recently published study also shows the superiority of baclofen compared to placebo administration while observing a sex × dose interaction effect with men having a greater benefit from high-dose and women from low-dose administration (Garbutt et al. 2021). As mentioned initially, there are mixed results and some of the studies showed no superior outcomes for baclofen (e.g., Colombo et al. 2004; Garbutt et al. 2010)). However, it should be taken into account that all studies varied regarding study designs, focuses, doses of baclofen, and differently severely affected patients, which makes the comparability difficult.

On the *neurobiological* level, the slot machine paradigm evoked robust brain responses during gain anticipation at *baseline (t1)* in bilateral striatal areas (especially in the putamen and NAcc), thalamus, and insula — areas known to be involved in reward processing (Haber and Knutson 2010; Liu et al. 2011; Lorenz et al. 2015a, b).

As an *effect of treatment*, we observed a significant decrease of insular activation at *t2* (re-test condition) in the BAC but not in the PLA group during gain anticipation. Diminished insula function has previously been shown to drastically reduce addictive behavior (Bechara 2001). Here, this reduced activation was observed during the performance of a slot machine task. A reduction in fronto-striatal activation during recurrent reward anticipation in a slot machine task has been previously observed in HC (Lorenz et al. 2015a, b); a typical re-test characteristic of HC as the authors interpreted this phenomenon indicating a neurobiological correlate of reduced *uncertainty and/or arousal* at repetition. This finding could be interpreted in the framework of the *incentive salience theory* (Robinson and Berridge 2000): repetition of the slot machine might decrease its relevance, i.e., salience. However, it might also reflect a possible *motivational lack* at re-test condition (Shao et al. 2013) and missing novelty by knowing the course of the task. In this context, it has been shown that even one training session before the scanning procedure reduced the striatal BOLD signal within the reward circuit such as ventral striatum and caudate nucleus in HC (Shao et al. 2013). Interestingly, in the current study, only patients treated with baclofen showed a significant desensitization effect (Lorenz et al. 2015a, b). This strengthens the interpretation that baclofen as a pharmacological aid might help to “normalize” those brain processes enabling AUD patients to flexibly adapt their reward anticipation and thus improve their adjustment during changing reinforcement contingencies (e.g., desensitization processes). Two other pharmaco-fMRI studies conducting perfusion fMRI during either acute baclofen administration (20 mg) or a daily dosage of baclofen (80 mg) in smokers also observed a blood flow reduction in the insula (and VS) in the treatment group, confirming baclofen’s effects on the reward circuitry (Franklin et al. 2011, 2012). This is further supported by our own previous study (Beck et al. 2018) and neuroimaging findings of Logge and colleagues, who observed reduced alcohol-related cue reactivity using fMRI and applying 75 mg baclofen per day (Logge et al. 2019). Of note, this reduction in fronto-striatal brain activity was not observed in the low-dose-treated group (30 mg baclofen). Thus, these and our present results indicate that higher dosages of baclofen might have a significant beneficial effect on reward processing in AUD patients. In accordance with our findings, Naqvi and colleagues (2010) suggested that “the insula is a critical neuronal substrate” in nicotine dependence and found in this regard that smokers with insular brain lesions were able to quit smoking easier than smokers with no structural damage in this region.

In the *exploratory analyses*, we observed a *correlation* between the degree of desensitization and baclofen blood serum level. A stronger decrease in insula activation from t1 to t2 was associated with higher levels of baclofen blood serum. This finding further corroborates the assumption that baclofen is involved in the modification of brain reward processes, especially in insular regions, putatively via effecting dopaminergic neurotransmission. Thus, one mechanism of action of baclofen in the treatment of AUD might be a GABAergic modulation of the dopaminergic neurotransmission within the mesolimbic reward system (Beck et al. 2018; Fadda et al. 2003). However, future (preclinical and human) studies are warranted to investigate in depth the specific modes of action of baclofen in AUD.

Although, our results extend the knowledge about the effects of baclofen in the treatment of AUD, some limitations of the present study need to be considered.

First, the small sample size limits the statistical power of our analyses, and the robustness of our results needs to be proven in replication studies. Although we observed statistically relevant differences between baclofen and placebo treatment and their respective neurobiological correlates in a longitudinal design, this observation requires replication in a larger sample. Since the here-included sample is a sub-sample of Müller et al. (being a preregistered clinical trial), we were not able to further increase the sample size (Müller et al. 2015). Secondly, our study design with individual high-dose baclofen differed from some other RCTs (except Beraha et al. 2016; Müller et al. 2015; Reynaud et al. 2017), which used fixed categories of dosages and mostly lower dosages (> 60 mg/daily) (for review, see de Beaurepaire (2018) and Pierce et al. (2018)), thus reducing comparability. Furthermore, it would be recommended that future studies examine the effect of assumptions regarding the study medication taken (BAC vs. PLA) in order to focus on efficacy expectancy. Thirdly, there were unspecific group differences regarding lifetime consumption of alcohol and anxiety at baseline (t1). However, there was no effect on the results when co-varying for those variables. Regarding anxiety, some studies showed that baclofen has anxiolytic effects which could have been a significant mediator in our sample. Although we did not see anxiety-specific effects (neither in assessment via questionnaire nor as a covariate in imaging analysis), future studies focusing on different (baseline) levels of anxiety are desirable to elucidate the current inconsistency in findings. Fourthly, although the used slot machine task as an ecologically valid passive reward task is known to evoke robust activations of the so-called reward system (Dreher et al. 2008; Lorenz et al. 2014, 2015a, b), it is strongly recommended that future research replicates our findings via performance-driven reward tasks as, e.g., monetary incentive delay tasks.

Lastly, due to the individual titration of the study medication, not all BAC patients reached the maximum dosage of 270 mg/d. Nevertheless, in flexible dosage studies like ours, it is common that the placebo group reaches the maximum dosage more likely (e.g., Reynaud et al. 2017: 88%, Müller et al. 2015: 67.9% of patients) compared to the intervention group (Reynaud et al. 2017: 67%; Müller et al. 2015: 35.7% of patients) (Müller et al. 2015; Reynaud et al. 2017), most likely due to the lack of effect of placebo medication and a tendency to therefore increase placebo dosage.

Taken together, the present neuroimaging results contribute to the understanding of underlying neurobiological mechanisms of (individually titrated) baclofen treatment, in particular within the domain of AUD-related altered reward anticipation processing.

Our results indicated that baclofen might desensitize right insula activation during recurrent reward processing in AUD patients after 2 weeks of individual high-dose baclofen treatment as well as increase the abstinence rate. Baclofen might thus enable a more flexible adaptation of neuronal activation in terms of repetition effects similar to HC, in accordance with the key role attributed to insula function in addiction (Droutman et al. 2015). Furthermore, observed correlations between baclofen dosage level in the blood and desensitization of the right insula support the dose-related effects of baclofen on the (dopaminergic) reward circuitry (Boehm et al. 2002).

## Supplementary Information

Below is the link to the electronic supplementary material.

Supplementary file1 (DOCX 14 KB)

## Data Availability

The participants of this study did not give written consent for their data to be shared publicly, so due to the sensitive nature of the research supporting data is not available.
