# Supplementary material for: Effects of baclofen on insular gain anticipation in alcohol-dependent patients — a randomized, placebo-controlled, pharmaco-fMRI pilot trial
Source: Psychopharmacology (Berl). 2022 Dec 20;240(1):171–83. doi: 10.1007/s00213-022-06291-6 (PMC9816215; doi:10.1007/s00213-022-06291-6)
Supplement: Supplementary file 1 — Supplementary file1 (DOCX 14 KB) [file 213_2022_6291_MOESM1_ESM.docx]

**Table 3** Effect of Task: Brain regions with covariates

| **Brain regions** | **L/R** | **k** | **MNI** | | | **t** | _FWE(cluster)_ |
| --- | --- | --- | --- | --- | --- | --- | --- |
|  |  |  | **x** | **y** | **z** |  |  |
| Putamen | L | 53 | -18 | 2 | -10 | 8.59 | <.001 |
| Putamen | R | 35 | 18 | 5 | -10 | 8.02 | <.001 |
| Thalamus | R | 14 | 9 | -22 | -4 | 7.83 | <.001 |
| Thalamus | L | 2 | -6 | -25 | -7 | 6.37 | .013 |
| Anterior Insula | R | 2 | 33 | 26 | 5 | 6.28 | .013 |
| Abbreviations: R=right and, L=left hemisphere; k=cluster size; x, y, z=MNI Montreal Neurobiological Institute space; t=t-value; p=p-value at statistical threshold of p<.05 family-wise error (FWE) whole brain corrected at cluster level and reported via SPM implemented brain atlas “Neuromorphometrics”. | | | | | | | |
